# Ginsenoside Rh2 represses autophagy to promote cervical cancer cell apoptosis during starvation

**DOI:** 10.1186/s13020-020-00396-w

**Published:** 2020-11-12

**Authors:** Jiawen Wang, Shuai Bian, Siming Wang, Song Yang, Wanying Zhang, Daqing Zhao, Meichen Liu, Xueyuan Bai

**Affiliations:** Jilin Ginseng Academy, Changchun University of Chinese Medicine, Boshuo Road 1035, Changchun, 130117 Jilin People’s Republic of China

**Keywords:** Cervical cancer, Apoptosis, Herbs, Cell signaling

## Abstract

**Background:**

Cancer cells through autophagy-mediated recycling to meet the metabolic demands of growth and proliferation. The steroidal saponin 20(S)-ginsenoside Rh2 effectively inhibits the growth and survival of a variety of tumor cell lines and animal models, but the effects of Rh2 on autophagy remain elusive.

**Methods:**

Cell viability was measured by CCK-8 (cell counting kit-8) assays. Apoptosis, ROS generation and mitochondrial membrane potential were analyzed by flow cytometry. Western blot analyses were used to determine changes in protein levels. Morphology of apoptotic cells and autophagosome accumulation were analyzed by DAPI staining and transmission electron microscopy. Autophagy induction was monitored by acidic vesicular organelle staining, EGFP-LC3 and mRFP-GFP-LC3 transfection. Atg7 siRNA and autophagy regulator was used to assess the effect of autophagy on apoptosis induced by G-Rh2.

**Results:**

In this study, we found that low concentration G-Rh2 attenuated cancer cell growth and induced apoptosis upon serum-free starvation. Caspase 3 inhibitors failed to block apoptosis in G-Rh2-treated cells, indicating a caspase-independent mechanism. G-Rh2-treated cells in serum-deprived conditions showed impaired mitochondrial function, increased release and nuclear translocation of apoptosis-inducing factor, but little changes in the mitochondrial and cytoplasmic distributions of cytochrome C. Annexin A2 overexpression in 293T cells inhibited G-Rh2-induced apoptosis under serum-starved conditions. Meanwhile, G-Rh2 reduced lysosomal activity and inhibited the fusion of autophagosome and lysosome, leading to a block of autophagic flux. Knockdown Atg7 significantly inhibited autophagy and triggered AIF-induced apoptosis in serm free condition. The autophagy inducer significantly decreased the apoptosis levels of G-Rh2-treated cells in serum-free conditions.

**Conclusions:**

Under nutrient deficient conditions, G-Rh2 represses autophagy in cervical cancer cells and enhanced apoptosis through an apoptosis-inducing factor mediated pathway.

## Background

*Panax ginseng* C.A. Meyer has been widely used in East Asian countries for thousands of years as a traditional medicine [1]. Ginsenosides extracted from ginseng display a wide range of pharmacological activities and several of these have been shown to inhibit cancer cell proliferation [2, 3]. There is increasing evidence that ginsenoside 20 (S)-Rh2 exerts antitumor effects in several cancer models [4–6].

Cervical cancer is one of the most prevalent cancer types affecting women worldwide [7]. The primary treatment options for cervical cancer include radiation and chemotherapy, both of which can be combined with herbal remedies [8]. Several ginsenosides exert cytotoxic activity in cervical cancer cells [9–12].

Autophagy captures intracellular components such as proteins and organelles and degrades them in lysosomes to sustain metabolism and cellular homeostasis [13]. Autophagy is generally recognized as a pro-survival mechanism in response to various stressors, including nutritional deficiency and chemotherapy [14, 15]. In some cancers, cells maintain mitochondrial function and energy homeostasis through autophagy-mediated recycling to meet the metabolic demands of growth and proliferation. Thus, inhibiting autophagy could cause substrate accumulation, cell growth arrest and cell death, all of which would serve to restrict tumor progression [16].

The effects of various ginsenosides on autophagy have been investigated [17–21], however, no reports have investigated the connection between autophagy and apoptosis in cervical cancer cells treated with G-Rh2. In this study, G-Rh2 enhanced susceptibility to serum deprivation-induced apoptosis via inhibiting autophagy. These results demonstrated a novel activity of G-Rh2, autophagy suppression, which enhanced apoptosis in HeLa cells under starvation conditions. These data suggested that G-Rh2 could be an alternative combination therapy strategy for cervical cancer patients.

## Materials and methods

### Cells and transfection

HEK293T (ATCC, CRL-11268), HeLa (ATCC, CCL-2), and C-33 A (ATCC, HTB-31) cells were cultured in DMEM supplemented with 10% fetal bovine serum (FBS) at 37 °C in a 5% CO_2_ environment. Lipofectamine 2000 (Invitrogen, Carlsbad, CA, USA) was used for transient plasmid and siRNA transfections. The sequences of the siRNA targeting Atg7 were 5′-GACAUUAAGGGUUAUUACUTT-3′and 5′-AGUAAUAACCCUUAAUGUCTT-3′. The control siRNA sequences were 5′-UUCUCCGAACGUGUCACGUTT-3′ and 5′-ACGUGACACGUUCGGAGAATT-3′.

### Cell counting kit-8 (CCK8) assays

Cells were seeded into 96-well culture plates. The following day, culture medium was replaced with 100 µl of serum-free medium or normal medium with dimethylsulfoxide (DMSO) or different compounds and incubated for 24 h. Before detection, CCK8 substrate was added into the plates and incubated for 1 h at 37 °C. Absorbance was measured at 450 nm using a microplate reader (Tecan Infinite 200PRO, Tecan, Mannedorf, Switzerland).

### Antibodies and reagents

The primary antibodies used in this study were as follows: anti-Tubulin mAb (BioLegend, San Diego, CA, USA), anti-p62 mAb, anti-LC3A/B mAb (Cell Signaling Technology, Danvers, MA, USA), anti-Cytochrome C mAb, anti-COX IV pAb, anti-Lamin B1 mAb, anti-AIF mAb, anti-CTSB pAb (Beyotime, shanghai, China). HRP-conjugated goat anti-mouse IgG and goat anti-rabbit IgG (Jackson Immunoresearch, West Grove, PA, USA) and Alexa Fluor 488-conjugated goat anti-rabbit IgG antibody (Molecular Probes, Waltham, MA, USA) were used for detection.

Ginsenoside 20 (S)-Rh2 was purchased from Chengdu Must Biotech (Chengdu, China). The Annexin V-FITC/PI apoptosis detection kit was purchased from BD Biosciences (Franklin Lakes, NJ, USA). Rhodamine 123, Ac-DEVD-CHO, DAPI, the nuclear and cytoplasmic protein extraction kit and cell mitochondria isolation kit were purchased from Beyotime. CCK-8 was purchased from BOSTER Biotech (Wuhan, China). Rapamycin and Bafilomycin A1 (BA1) were purchased from InvivoGen (San Diego, CA, USA).

### Plasmid construction

The LC3B gene was obtained by PCR from cDNA of HeLa cells and subsequently subcloned using standard molecular biology procedures into the pEGFP-C3. The primers: forward 5′-GGAATTCTATGCCGTCGGAGAAGAC-3′ and reverse 5′-CGGGATCCTTACACTGACAATTTCAT-3′. Annexin A2 fragments were amplified from HeLa cDNA by PCR and subcloned into the VR1012 vector with an HA tag added in frame at its N-terminus. The primers: forward 5′-ATGTCTACTGTTCACGAAATCC-3′ and reverse 5′-GTCATCTCCACCACACAGG-3′. HA tag primers: forward 5′-CGTCTAGAGCCACCATGTCTACTGTTCACGAA-3′ and reverse 5′-CGGGATCCTCAAGCATAGTCTGGGACGTCGTATGGGTAGTCATCTCCACCACA-3′. GFP-mRFP-LC3, CD317 IHA, and VR1012 and pEGFP-N3 were described previously [22, 23].

### Apoptosis measurement

Cells were double-stained with propidium iodide (PI) and Annexin V-FITC using an apoptosis detection kit for flow cytometry. The samples were analyzed using a FlowSight^®^ Imaging Flow Cytometer (Merck Millipore, Burlington, MA, USA) and the software IDEAS Application V6.1.

### Mitochondrial membrane potential (MMP) measurement

Cells were stained with 1 µM rhodamine 123 for 30 min at 37 °C. After washing with ice-cold phosphate-buffered saline (PBS), the cells were acquired and analyzed by flow cytometry.

### Immunofluorescence

Cells were then fixed with 4% formaldehyde, permeabilized with 0.5% Triton X-100, and then blocked with 10% FBS. Immunostaining was performed by incubating cells with primary antibody and Alexa Fluor 488-conjugated IgG antibody. After DAPI staining, cells were observed using a fluorescence microscope with a 20× objective lens.

### Western blotting

Cells were harvested and resuspend with lysis buffer, boiled for 15 min, separated by SDS- PAGE, and then transferred onto nitrocellulose membranes (Whatman, Maidstone, UK). After blocking in 5% nonfat milk, membranes were probed with primary and secondary antibodies. Immunoreactivity was visualized by chemiluminescence. ImageJ software was used for densitometric analysis.

### Transmission electron microscopy assay

Cells were treated as indicated and fixed in 4% glutaraldehyde at 4 °C overnight. After dehydration, ultrathin sections were embedded and stained with uranyl actate/lead citrate. Images were captured under a transmission electron microscope (H-7650, Hitachi, Japan).

### Acidic vesicular organelle staining

After rinsing in PBS and fixing with 4% paraformaldehyde for 10 min, the cells were stained for acidic vesicular organelles in the dark for 30 min. Then, the cells were observed by fluorescence microscopy at 488 nm and imaged.

### Statistical analysis

All data represent at least 3 independent experiments and are expressed as mean ± SEM. Statistical comparisons were made by one-way analysis of variance and Dunnett’s post hoc test. *p < 0.05; **p < 0.01; ***p < 0.001; ns > 0.05.

## Results

### G-Rh2 repressed cancer cells proliferation and promoted apoptosis under serum-free conditions

**Fig. 1:**
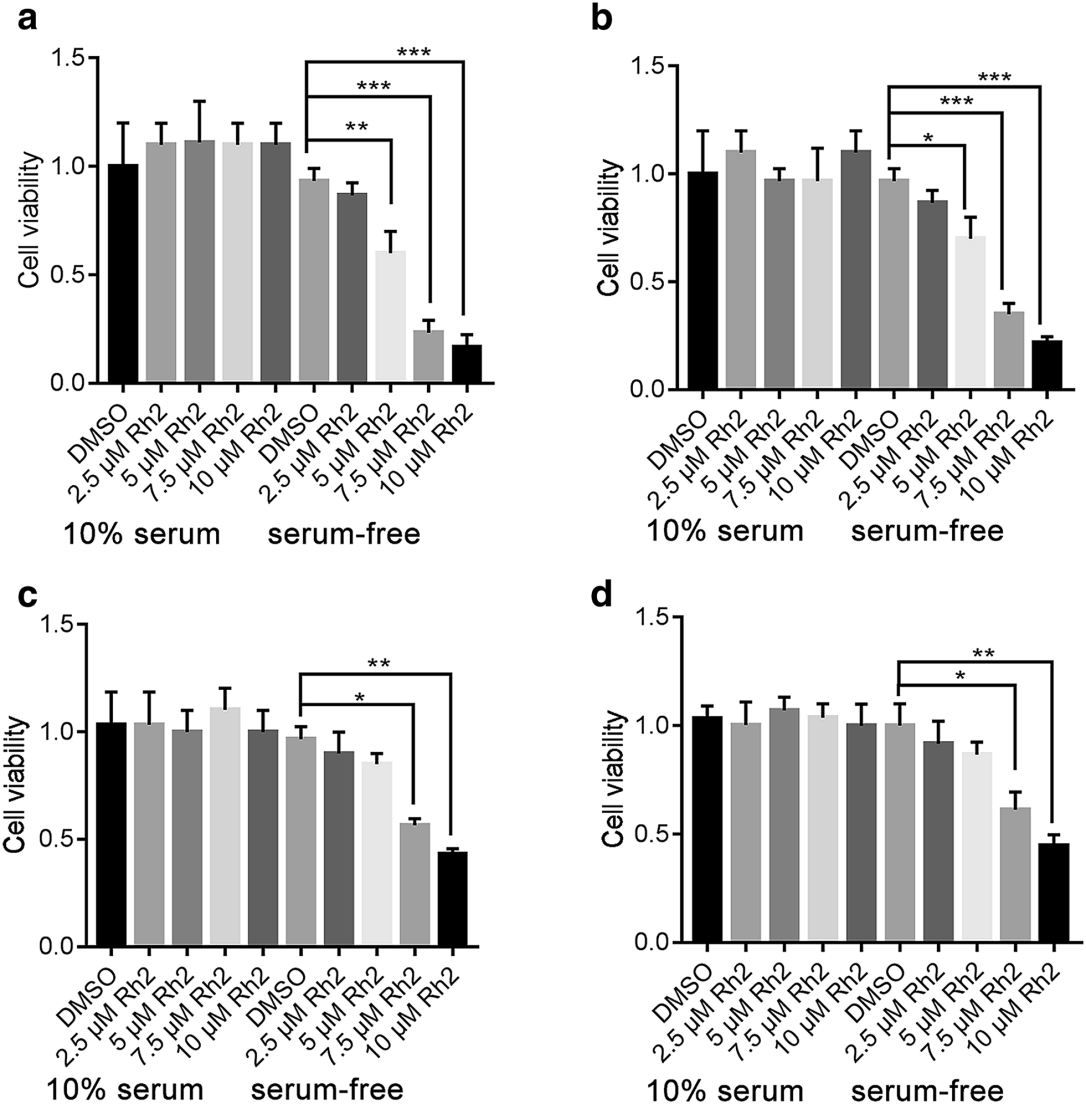
G-Rh2 represses cervical cancer cell proliferation under serum-starved conditions. CCK8 cell proliferation analysis of HeLa (**a**), C-33A (**b**), HEK293T (**c**), HUVEC (**d**) cells with different G-Rh2 concentrations under normal or serum-deprived conditions for 24 h.*p < 0.05, **p < 0.01, ***p < 0.001

G-Rh2 has been shown to have antitumor effect on a variety of cancer cells, including cervical cancer cells. Serum deprivation is often used as a means of emulating the tumor microenvironment. Here, we investigated the cytostatic activity of G-Rh2 during serum deprivation and normal conditions. As shown in Fig. 1, in normal conditions, G-Rh2 (0–10 µM) had no effect on cell proliferation. 5, 7.5 and 10 µM G-Rh2 significant inhibited cancer cells proliferation under serum-free conditions (Fig. 1a and b, Additional file 1: Figure S1B and C). Meanwhile, the cell viability of normal cells (293T and HUVEC) treated with 5 µM G-Rh2 were no obvious difference compared with control group (Fig. 1c and d). These data suggested that low concentration G-Rh2 exerted strong cytostatic activity under serum-free conditions.

**Fig. 2:**
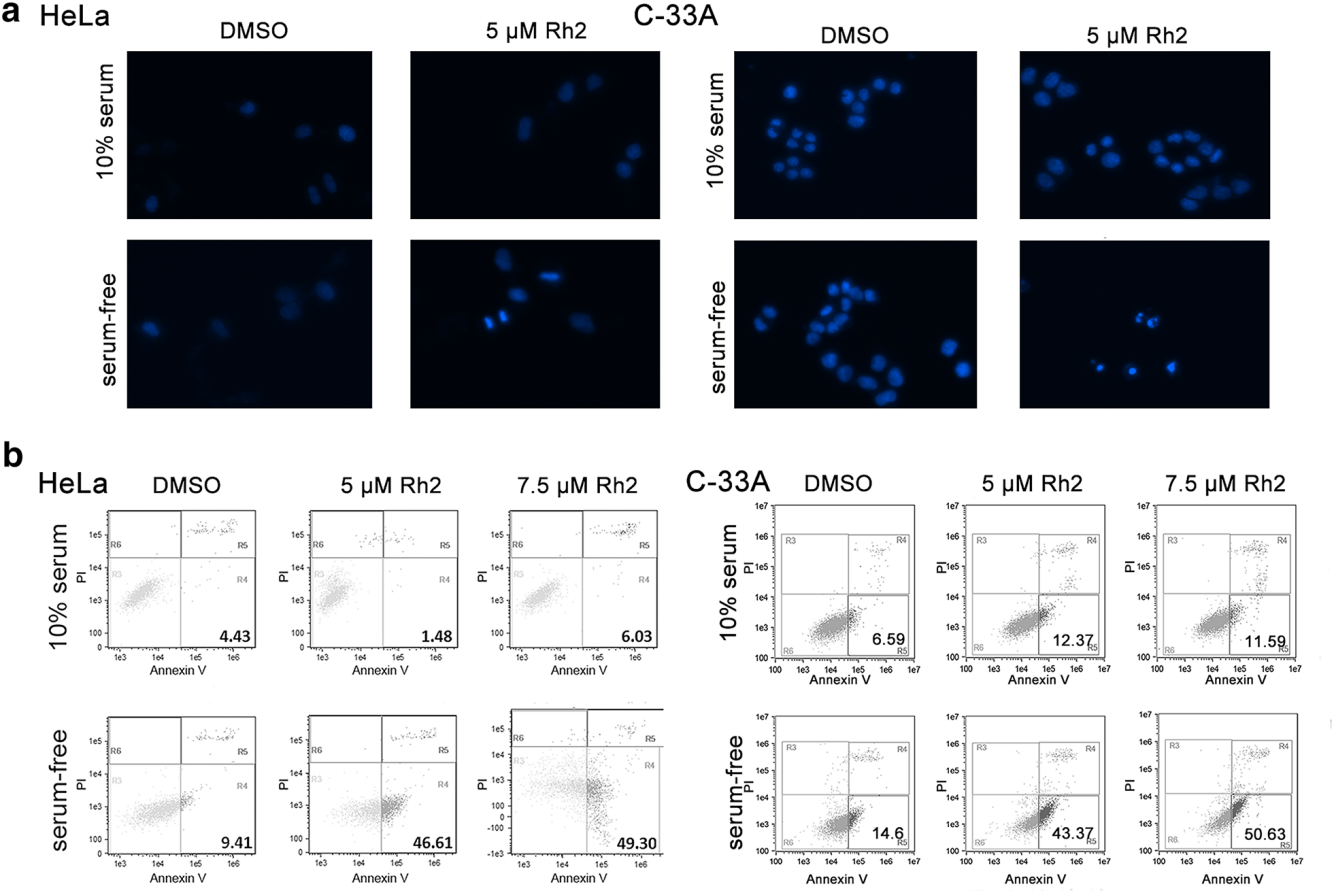
G-Rh2 enhances cervical cancer cell apoptosis under serum-deprived conditions. **a** Treated cells were fixed and stained with DAPI and observed under a fluorescence microscope. **b** Flow cytometric analyses of apoptotic cells with different G-Rh2 concentrations under normal or serum-deprived conditions for 24 h

To determine whether the decrease in cell viability was caused by apoptosis, we used Hoechst 33342 staining and Annexin V/PI analysis. Figure 2a shows chromatin condensation and nuclear fragmentation in HeLa and C-33A cells treated with G-Rh2. As showed in Fig. 2b (Additional file 2: Table S1), G-Rh2 did not affect apoptosis in cancer cells cultured in normal medium, but markedly enhanced apoptosis during 24 h serum deprivation. Moreover, in serum-free conditions, 7.5 µM G-Rh2 caused a decrease in the percentage of S and G0/G1 phase cells (Additional file 1: Fig. S1D, Additional file 3).

### G-Rh2 impaired mitochondrial function resulting in AIF release and nuclear accumulation

**Fig. 3:**
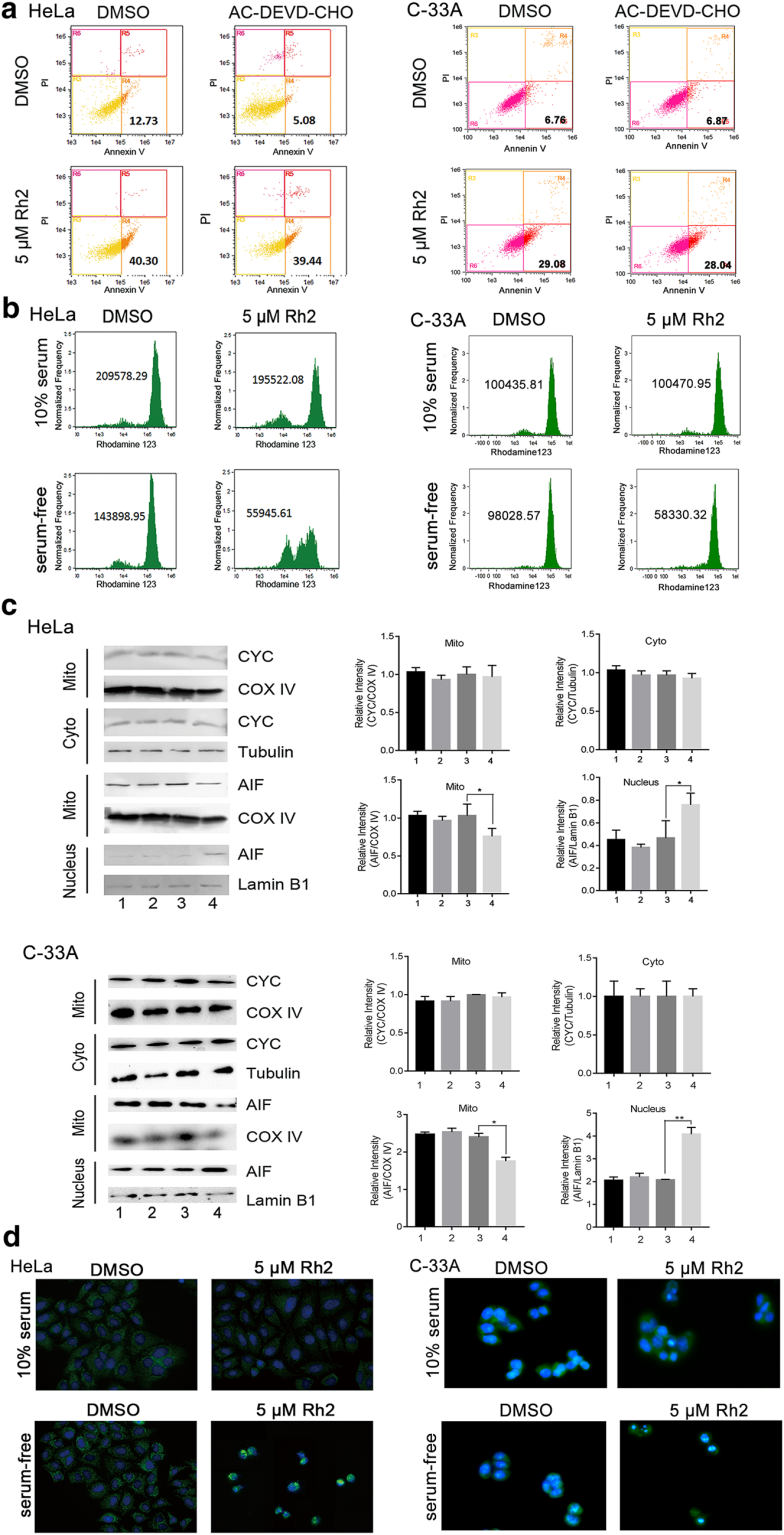
G-Rh2 regulates apoptosis through the mitochondrial-AIF pathway under serum-starved conditions. **a** Flow cytometric analysis of apoptotic HeLa and C-33A cells with DMSO or G-Rh2 under serum-free conditions in the presence or absence of 40 μM Ac-DEVD-CHO. **b** Flow cytometry in combination with Rhodamine 123 staining performed in HeLa and C-33A cells. Cells were treated with DMSO or G-Rh2 in the presence or absence of serum. **c** Subcellular distribution of cytochrome c and AIF in HeLa and C-33A cells. Cells were treated with DMSO or G-Rh2 in the presence or absence of serum for 24 h, and then collected for subcellular fractionation. COX IV, Tubulin, and Lamin B1 served as controls for mitochondria, cytoplasm, and nuclear fractions, respectively. Lane 1, 10% FBS and DMSO; lane 2, 10% FBS and 5 μM G-Rh2; lane 3, serum-free and DMSO; lane 4, serum-free and 5 μM G-Rh2. **d** Immunofluorescence analysis of AIF in cervical cancer cells. HeLa and C-33A cells were treated with DMSO or G-Rh2 in the presence or absence serum. DAPI was used for nuclear staining. G-Rh2 promotes apoptosis through an autophagy-dependent mechanism. Flow cytometric analyses of apoptotic HeLa cells with DMSO or G-Rh2 under serum-free conditions in the presence or absence of 100 nM BA1, 500 nM Rapamycin, or 5 mM 3-MA

To investigate the apoptosis pathway, we employed Ac-DEVD-CHO, a specificity caspase 3 inhibitor. The inhibitor reduced serum deprivation-induced apoptosis but no significant effect on the apoptosis induced by G-Rh2 (Fig. 3a, Additional file 3: Table S1). Mitochondria are the primary organelles that induce apoptosis [24]. We compared the levels of mitochondrial membrane potential (MMP) between DMSO- and G-Rh2-treated using by Rhodamine 123. Flow cytometric analysis showed that G-Rh2 had little effect on mitochondrial function in normal conditions but prominently reduced MMP in serum-deprived cells (Fig. 3b). Next, we detected the distribution of apoptosis-inducing proteins (AIF) and cytochrome c. As shown in Fig. 3c and d, the level of nuclear AIF was significantly higher in G-Rh2-treated cells from serum-deprived conditions than in the DMSO controls. This nuclear accumulation was accompanied by a decline in mitochondrial distribution, suggesting that G-Rh2 enhanced AIF release and nuclear translocation in serum-free cells. Meanwhile, the distribution of cytochrome c in the mitochondria and cytoplasm had no visible change in any conditions. Together, these data suggested that G-Rh2 promotes serum deprivation-induced apoptosis through the AIF pathway.

### G-Rh2 inhibited starvation-induced autophagic flux and lysosomal activity

**Fig. 4:**
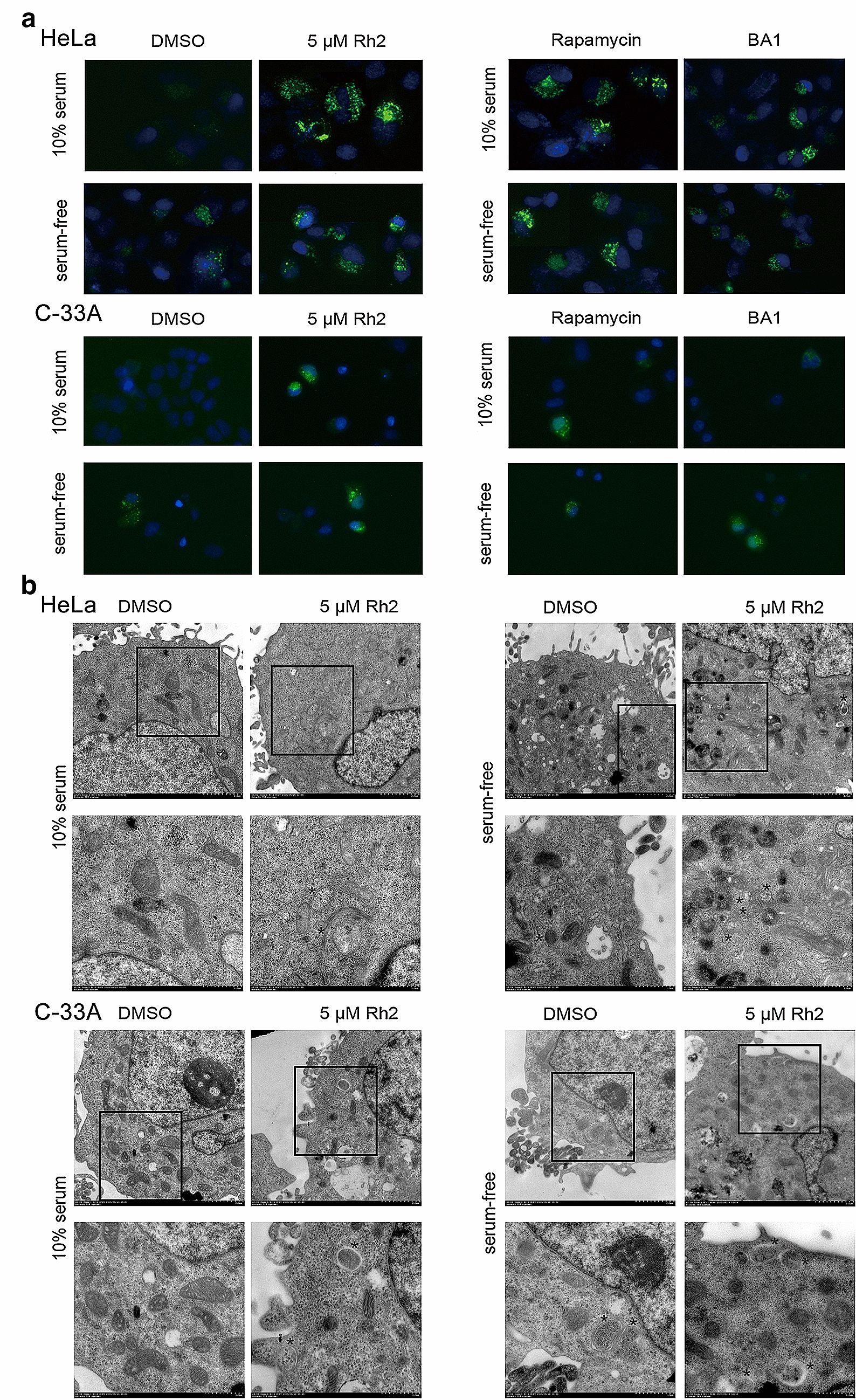
G-Rh2 regulated autophagy in cervical cancer cells. **a** HeLa and C-33A cells were transfected with GFP-LC3B, and after 24 h the cells were treated with DMSO, G-Rh2, Rapamycin or BA1 in normal or serum-free medium. After 24 h, the cells were observed with a fluorescence microscope. **b** HeLa and C-33A cells were treated with DMSO or G-Rh2 in normal or serum-free medium for 24 h, fixed and examined using transmission electron microscopy. Higher power magnification of the image of cells revealed autophagosomes (asterisk)

According to recent report, an autophagy modulator scoring system (AMSS) was designed to evaluate methodological integrity [25, 26]. To verify whether G-Rh2 participates in autophagy, we examined the effect of G-Rh2 on autophagy by measuring GFP-LC3B puncta formation. The results revealed that G-Rh2 obviously increased the number of LC3B puncta in normal and starving HeLa cells and C-33A cells (Fig. 4a). Transmission electron microscopy was used to observe the accumulation of autophagic vacuoles in G-Rh2-treated cells, which revealed an increased number of autophagic vacuoles compared with control cells (Fig. 4b).

**Fig. 5:**
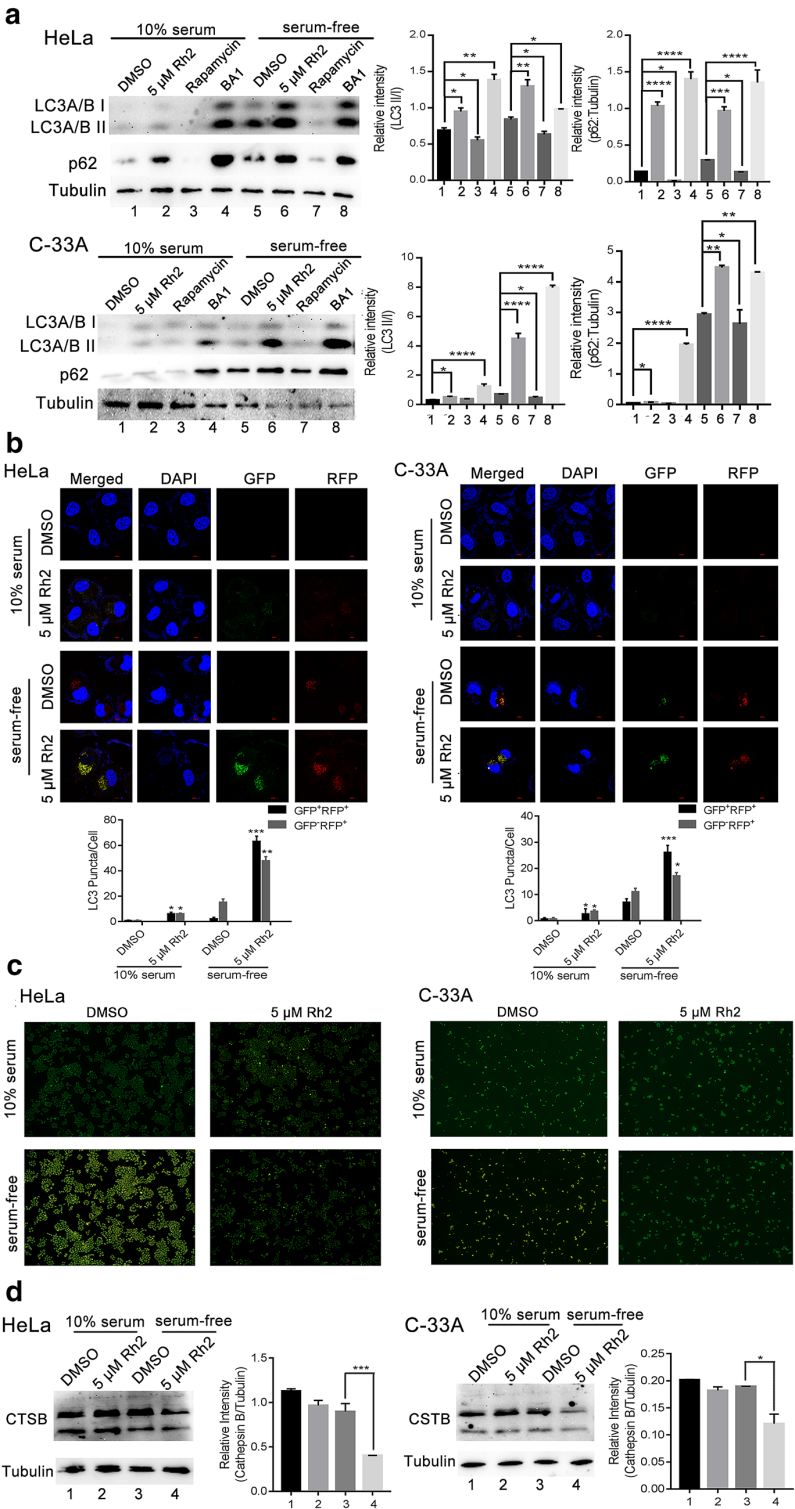
G-Rh2 represses starvation-induced autophagic flux. **a** HeLa and C-33A cells were treated with DMSO or G-Rh2 or autophagy regulators in the presence or absence of serum and were harvested at 24 h. Whole cell lysates were prepared and analyzed for p62 and LC3, tubulin served as the loading control. **b** HeLa and C-33A cells were transiently transfected with mRFP- GFP-LC3 and cells were treated with DMSO or G-Rh2 in the presence or absence of serum and harvested for 24 h. At the end of treatment, cells were observed for the change of both green and red fluorescence using a confocal microscope. **c** Acidic vesicular organelles were examined by incubating HeLa and C-33A cells with acridine orange followed by fluorescence microscopy. **d** After HeLa and C-33A cells were treated with DMSO or G-Rh2, the expression of CTSB was determined by western blotting

An increase in LC3 II in response to a drug may represent either increased generation of autophagosomes or a block in autophagosomal maturation and degradation. Meanwhile, a reduction of substrate protein p62 can be regarded as a marker for increased autophagic flux. Western blot results showed that LC3 II and p62 protein levels were increased following G-Rh2 treatment (Fig. 5a, lanes 2 and 6). Rapamycin strongly induced autophagy leading to significant degradation of LC3 and p62 (Fig. 5a, lanes 3 and 7). The autophagy inhibitor BA1 prevented maturation of autophagic vacuoles and increased LC3 and p62 levels (Fig. 5a, lanes 4 and 8). The effect of G-Rh2 was similar to autophagy inhibitor BA1. In addition, we used HeLa and C-33A cells transfected with the tandem fluorescent-tagged LC3B (mRFP-GFP-LC3B) plasmid. RFP and GFP exhibit differing pH sensitivity, wherein GFP is quantitatively quenched in acidic compartments while RFP is stable. Therefore this probe capitalizes on the pH difference between the acidic autolysosome and the neutral autophagosome to monitor flux of LC3B from autophagosomes (RFP-positive GFP-positive; yellow dots) to autolysosomes (RFP-positive GFP-negative; red dots). Nutrient starvation treatment only resulted in the production of large amounts of red puncta. In the cells treated with G-Rh2, we observed the enhanced formation of yellow puncta. The results suggested that upon G-Rh2 treatment the autophagosomes do not fuse with lysosomes or that lysosomal function is impaired (Fig. 5b).

Because the autophagic flux depends on low pH, we analyzed the lysosomal pH by an acridine orange (AO) assay. AO is a fluorescent nucleic acid dye that accumulates in acidic spaces such as lysosomes. Under the low pH conditions in lysosomes, the dye emits red light when excited by blue light. Compared to the control group with serum, the DMSO group without serum showed an increased red signal, indicating that the pH value was decreased and autophagy was promoted, whereas the addition of G-Rh2 reduced the red signal, indicating inhibition of autophagy (Fig. 5c). The lysosome enzyme like Cathepsin B (CTSB) was used to examine the lysosome function. Western blotting assay demonstrated that G-Rh2 remarkedly downregulated the total and mature from protein level of CSTB in HeLa and C-33A cells (Fig. 5d). These data suggested that in Rh2-treated cells, the increased LC3 II levels and formation of GFP-LC3 puncta were not due to enhanced autophagy, but due to reducing lysosomal activity, inhibiting the fusion of autophagosome and lysosome, leading to a block of autophagic flux.

### Apoptosis induced by G-Rh2 was associated with autophagy

**Fig. 6:**
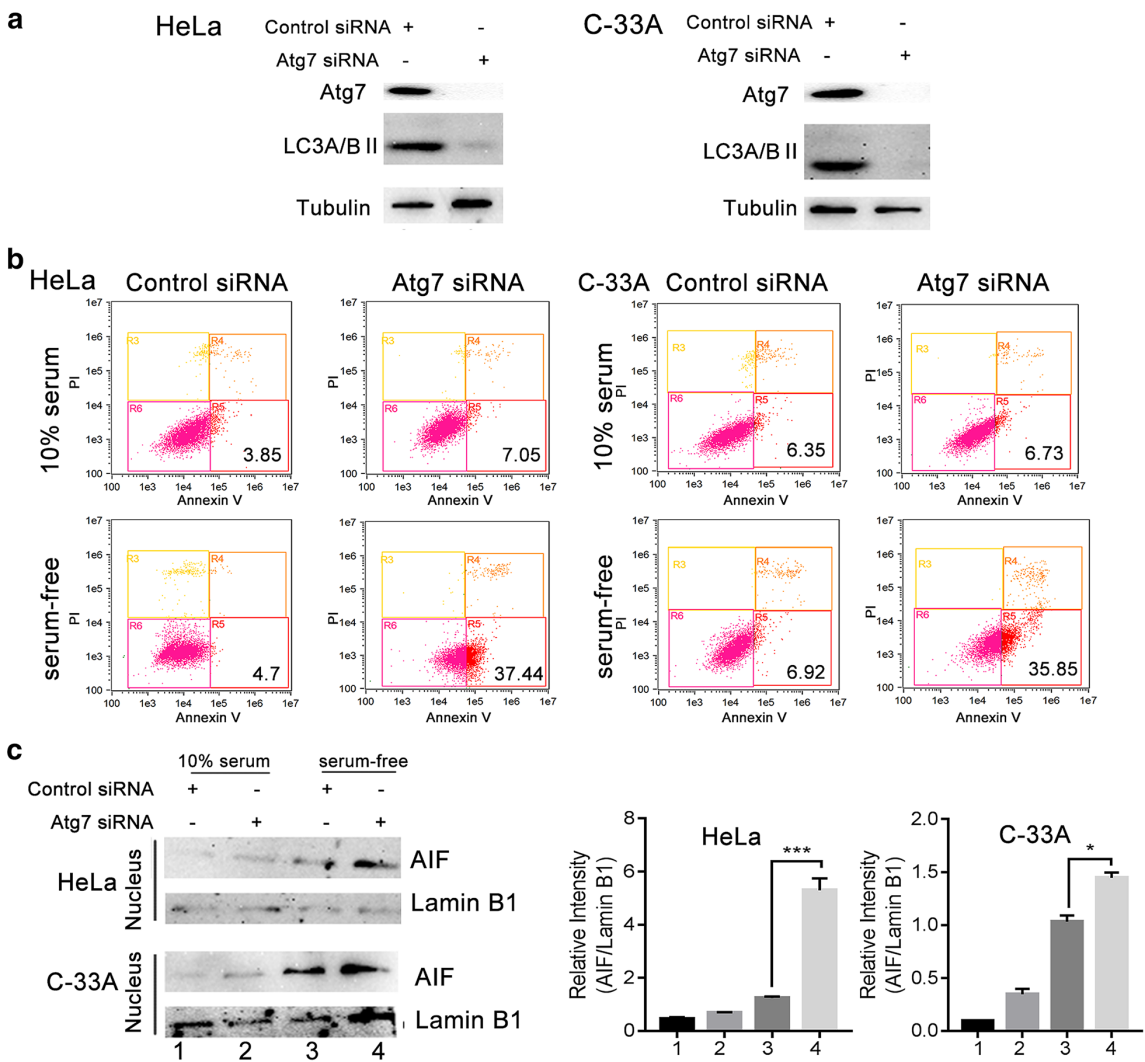
Silencing of Atg7 enhanced cell apoptosis and AIF nuclear translocation under serum-starved condition. **a** The autophagy inhibition effect of siAtg7 was detected by western blot. **b** Flow cytometry was used to detect the apoptosis of cells transfected with sicontrol or siAtg7 in the presence or absence of serum. **c** The effect of knockdown siAtg7 in expression level of AIF in nuclear. HeLa and C-33A cells were transfected with sicontrol or siAtg7, after 24 h the cells were cultured in the presence or absence of serum for another 24 h, and then collected for nuclear fractionation. The expression of AIF was determined by western blotting

To determine whether autophagy regulation was responsible for G-Rh2-induced apoptosis, we designed a siRNA to knockdown the expression of Atg7. As shown in Fig. 6a, the expression of Atg7 was downregulated in siAtg7 group and the sicontrol has no effect on the expression of Atg7. Meanwhile, the expression level of LC3 II was significantly reduced in the siAtg7 group. These results suggested the autophagy was inhibited in the cells transfected siAtg7. Then, for examine whether autophagy inhibition in serum free condition will trigger AIF-induced apoptosis, we detected the apoptosis rate and expression level of AIF in nuclear in the cells transfected siAtg7. As shown in Fig. 6b (Additional file 3: Table S1), knockdown Atg7 remarkably promoted apoptosis in serum free condition. According to the western blotting results, the level of nuclear AIF was higher in cells transfected siAtg from serum-deprived conditions than in the cells transfected sicontrol (Fig. 6c). In addition, autophagy inhibitors and G-Rh2 had additive effects on HeLa cell apoptosis during serum deprivation. Treatment with the autophagy agonist rapamycin significantly rescued G-Rh2-induced apoptosis in serum-free conditions (Additional file 4: Fig. S2). These data suggested that autophagy plays an important role in G-Rh2-induce apoptosis under serum-deprived conditions.

### Annexin A2 overexpression inhibited G-Rh2-induces apoptosis in serum-starved HEK293T cells

**Fig. 7:**
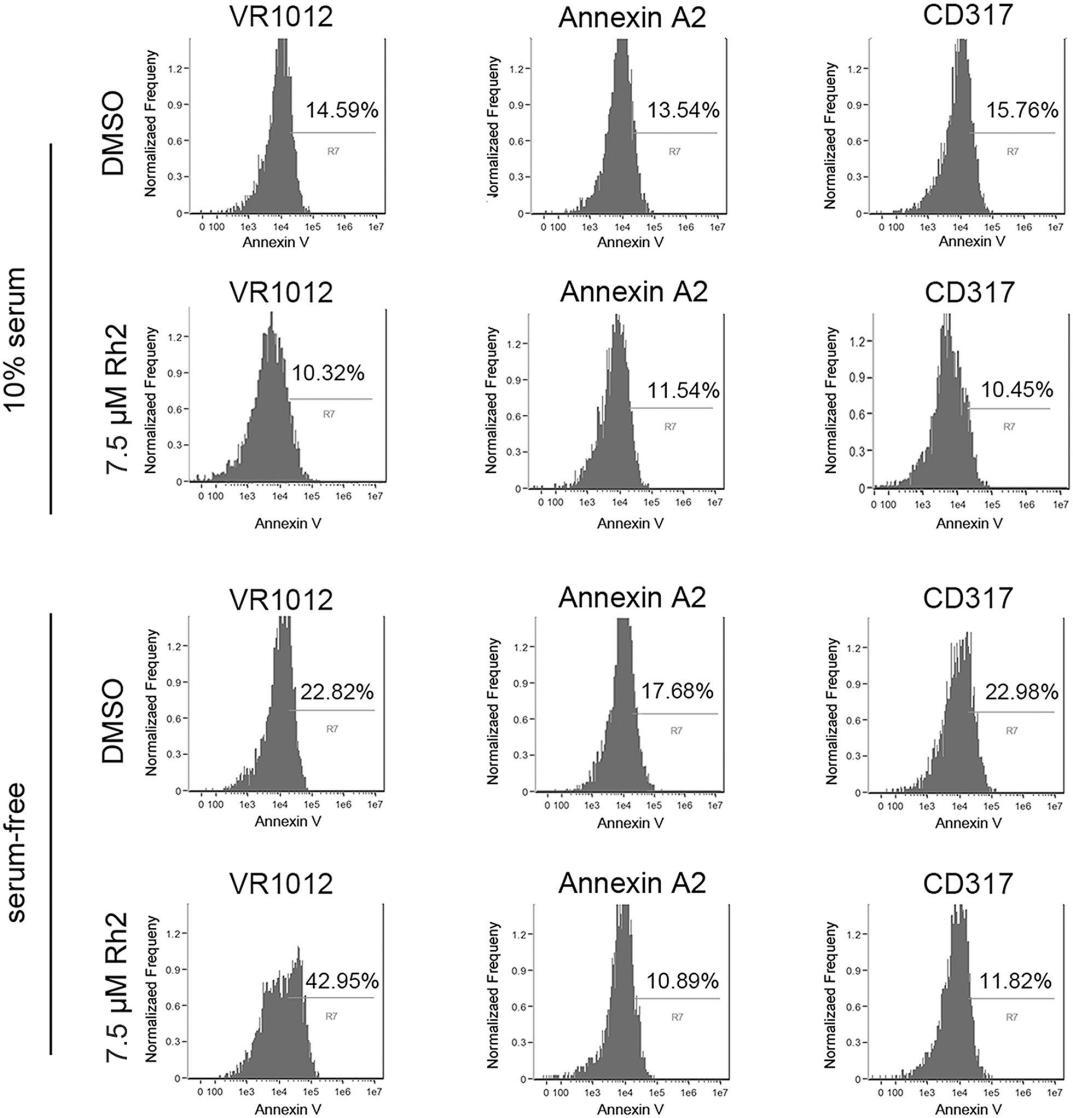
Annexin A2 and CD317 overexpression repress G-Rh2-induced apoptosis. HEK293T cells were transfected with GFP, Annexin A2 or CD317 expression plasmids, and after 24 h the cells were treated with DMSO or G-Rh2 in the presence or absence of serum. After another 24 h, flow cytometric analysis of apoptotic cells was performed

Annexin A2, is a member of the Annexin family that plays multiple cellular biological roles [27, 28]. Recently report claimed that 20(S) G-Rh2 directly bound Annexin A2 and promoting apoptosis in tumor cells [29]. To address whether Annexin A2 suppressed apoptosis following G-Rh2 treatment and serum deprivation, we forced Annexin A2 expression in HEK293T and detected its influence on apoptosis. CD317 is thought to protect cancer cells against serum deprivation-induced apoptosis, and was used as a positive control in this experiment [30]. The results showed that Annexin A2 overexpression did not affect apoptosis rates in serum-starved DMSO treated cells, but significantly suppressed apoptosis in G-Rh2-treated cells that were serum-deprived (Fig. 7 and Additional file 4: Figure S2B). This may be due to an excess of Annexin A2 binding to G-Rh2 and hindering G-Rh2 activity, or that expression of the protein improves starvation- induced autophagy and reduces apoptosis.

## Discussion

**Fig. 8:**
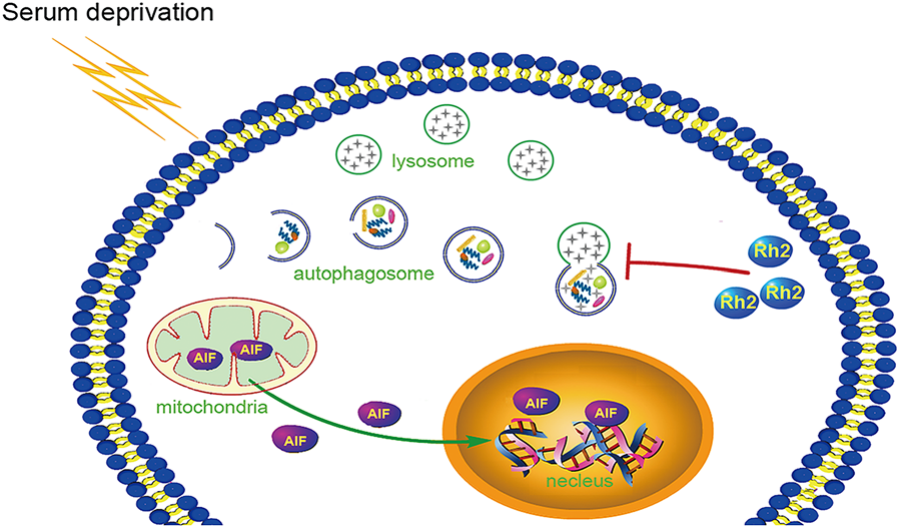
Schematic model

Previous reports have shown that the higher autophagy levels are correlated with worse prognosis in cancer patients [31]. Thus, inhibiting autophagy has been accepted as a promising therapeutic strategy in combination with existing cancer therapies. In this study, we conducted in vitro experiments to investigate the role of G-Rh2 in cervical cancer cell lines under serum-free conditions. These data demonstrated that G-Rh2 inhibited serum deprivation-induced autophagy and promoted apoptosis through the mitochondria-AIF pathway (Fig. 8).

Rh2 is a key molecule in ginseng extracts that exhibits significant anticancer effects in cervical cancer cells. It has been reported that Rh2 and betulinic acid association could increase caspase-8 activity [12], adjust AKT/GSK3β pathway [32], increase p53 expression [33], and induce apoptosis of cervical cancer cells. In this study, compared with cells in normal medium, lower concentration G-Rh2 significantly reduced cervical cancer cells proliferation and promoted apoptosis in serum-free medium. To date, studies have revealed three major apoptotic pathways: the mitochondrial pathway (intrinsic pathway), death receptor pathway (external pathway), and endoplasmic reticulum stress pathway [34, 35]. Here, we found that G-Rh2 increased ROS generation, mitochondrial membrane degradation, AIF release and nuclear translocation, and then induced mitochondrial-mediated apoptosis in serum-free cells.

Autophagy is a catabolic process that is conserved across eukaryotes. From regulating basic metabolic functions in cells to various pathological conditions, autophagy has become a central regulation point that controls energy homeostasis. Cells activate autophagy under extreme conditions to relieve various stresses. Several reports have considered that G-Rh2 could induce autophagy during induced apoptosis in cancer cells [35–37]. However, in this study, lower concentration G-Rh2 reduced lysosomal activity, inhibiting the fusion of autophagosome and lysosome blocked of autophagic flux in cervical cancer cells and increased apoptosis in starvation conditions. There are two possible explanations for these results: (1) G-Rh2 could regulate autophagy in both directions with different doses leading to different results, or (2) G-Rh2 could have different effects in different tumors. Lower concentration G-Rh2 could not directly induce apoptosis in cancer cells, but by inhibiting autophagy, impaired the self-regulatory capacity of HeLa cells during nutritional deprivation. Then, G-Rh2-treated serum-deprived cells enhanced MMP loss and AIF release and nuclear translocation, all of which increased apoptosis.

## Conclusion

In this study, we found that low concentration G-Rh2 attenuated cancer cell growth and induced apoptosis upon serum-free starvation. G-Rh2 is a new autophagy inhibitor that has potential clinical and research value. G-Rh2 could be used in combination therapy with a nutritional blockade (interventional therapy) or chemotherapeutic drug that causes autophagy in treated tumors. The effect of different G-Rh2 concentrations on autophagy levels in different cancers will be part of our future research plans.

## Supplementary information

**Additional file 1: Fig. S1** (A) Cytotoxicity screenings of G-Rb1, G-Rh2 and G-Rd in HeLa cells under normal or serum-deprived conditions. CCK8 cell proliferation analysis of A549 (B) / B16(C) cells with different G-Rh2 concentrations under normal or serum-deprived conditions for 24 h. (D) Flow cytometric analyses of the cell cycle distribution of HeLa and C-33A cells with different G-Rh2 concentrations under normal or serum-deprived conditions for 24 h.

**Additional file 2: Table S1.** Statistical analysis of apoptosis, as determined by the flow cytometric evaluation to Figs. 2b, 3a, 3b, 6b and S2A.

**Additional file 3.** Supplement of materials and methods.

**Additional file 4: Fig. S2** (A) G-Rh2 promotes apoptosis through an autophagy-dependent mechanism. Flow cytometric analyses of apoptotic HeLa cells with DMSO or G-Rh2 under serum-free conditions in the presence or absence of 100 nM BA1, 500 nM Rapamycin, or 5 mM 3-MA. (B) Statistical analysis of apoptosis, as determined by the flow cytometric evaluation corresponding to Fig. 7. (C) Flow cytometric analysis of apoptotic HeLa cells with different G-Rh2 concentrations under normal or serum-deprived conditions for 48 h.

## Data Availability

The datasets used in this study are available from the corresponding author upon reasonable request.

## Abbreviations

G-Rh2: 20(S)-Ginsenoside Rh2
CCK-8: Cell counting kit-8
FBS: Fetal bovine serum
DMSO: Dimethylsulfoxide
BA1: Bafilomycin A1
3-MA: 3-Methyladenine
PI: Propidium iodide
MMP: Mitochondrial membrane potential
PBS: Phosphate-buffered saline
DAPI: 4’,6-Diamidino-2-phenylindole
ROS: Reactive oxygen species
