# Supplementary figures and images for: Ginsenoside Rh2 represses autophagy to promote cervical cancer cell apoptosis during starvation
Source: Chin Med. 2020 Nov 12;15:118. doi: 10.1186/s13020-020-00396-w (PMC7661217; doi:10.1186/s13020-020-00396-w)

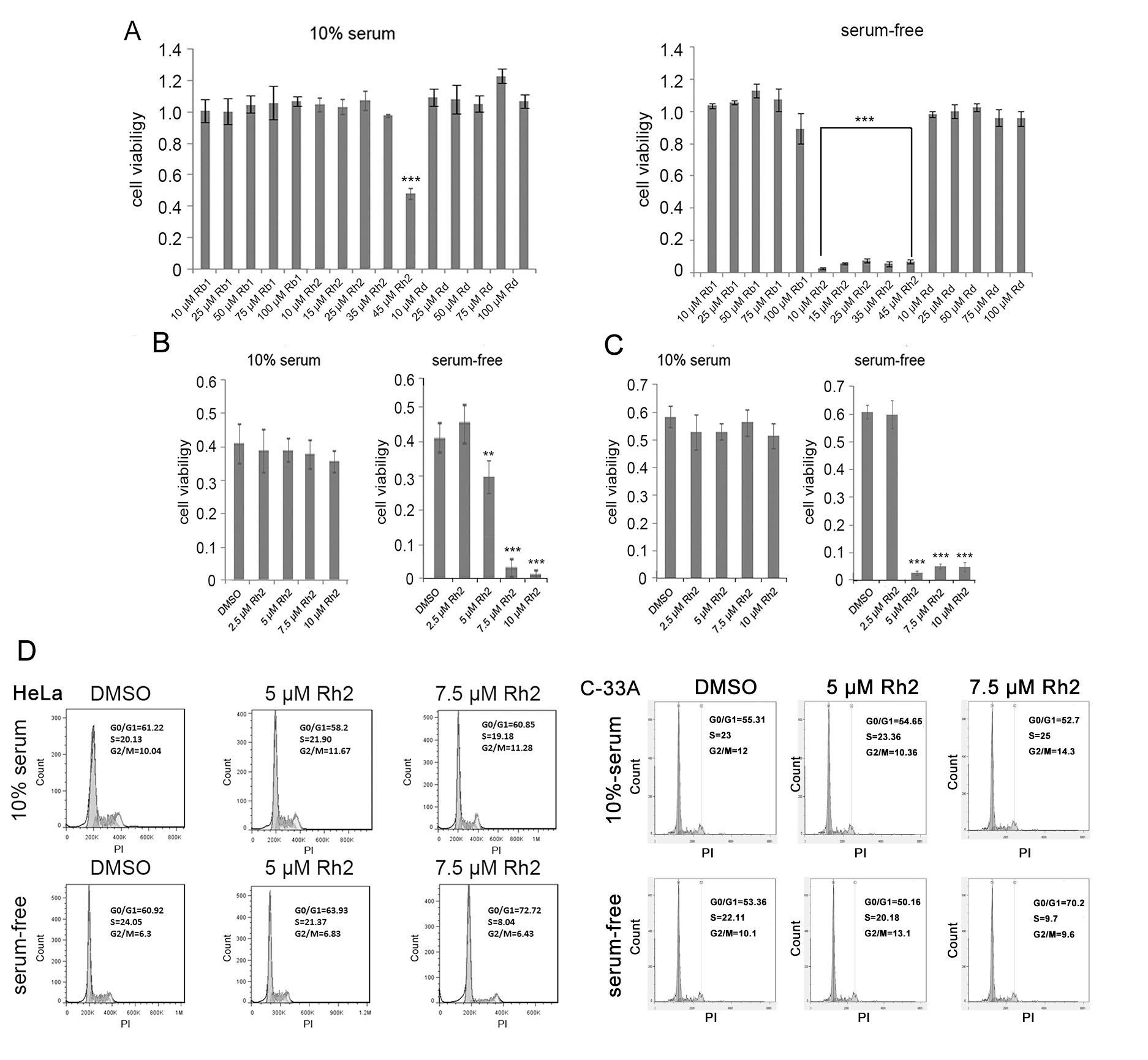

Supplement: Supplementary file 1 — Additional file 1: Fig. S1 (A) Cytotoxicity screenings of G-Rb1, G-Rh2 and G-Rd in HeLa cells under normal or serum-deprived conditions. CCK8 cell proliferation analysis of A549 (B) / B16(C) cells with different G-Rh2 concentrations under normal or serum-deprived conditions for 24 h. (D) Flow cytometric analyses of the cell cycle distribution of HeLa and C-33A cells with different G-Rh2 concentrations under normal or serum-deprived conditions for 24 h. [file 13020_2020_396_MOESM1_ESM.tif]

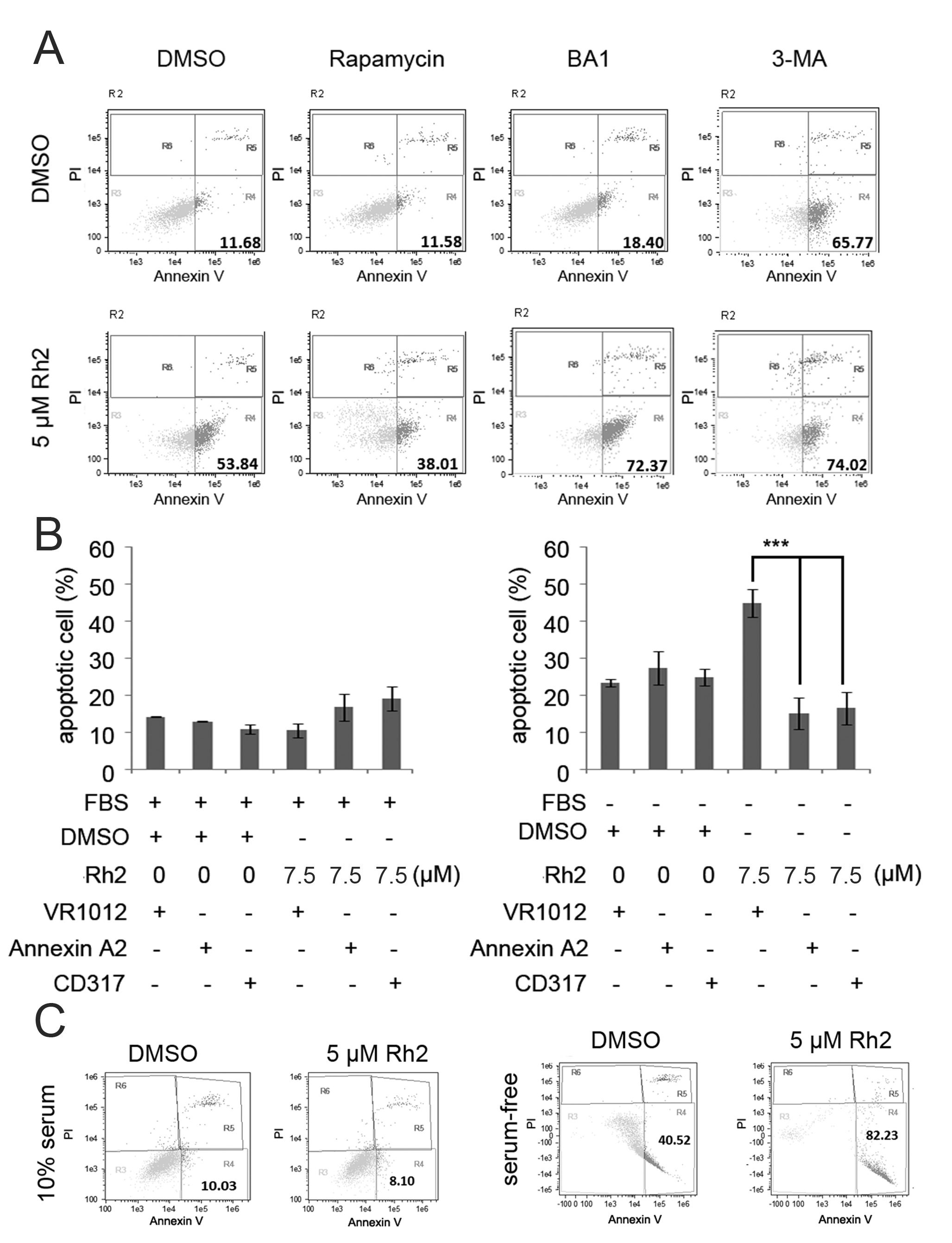

Supplement: Supplementary file 4 — Additional file 4: Fig. S2 (A) G-Rh2 promotes apoptosis through an autophagy-dependent mechanism. Flow cytometric analyses of apoptotic HeLa cells with DMSO or G-Rh2 under serum-free conditions in the presence or absence of 100 nM BA1, 500 nM Rapamycin, or 5 mM 3-MA. (B) Statistical analysis of apoptosis, as determined by the flow cytometric evaluation corresponding to Fig. 7. (C) Flow cytometric analysis of apoptotic HeLa cells with different G-Rh2 concentrations under normal or serum-deprived conditions for 48 h. [file 13020_2020_396_MOESM4_ESM.tif]
