# Supplementary material for: Ginsenoside Rh2 represses autophagy to promote cervical cancer cell apoptosis during starvation
Source: Chin Med. 2020 Nov 12;15:118. doi: 10.1186/s13020-020-00396-w (PMC7661217; doi:10.1186/s13020-020-00396-w)
Supplement: Supplementary file 2 — Additional file 2: Table S1. Statistical analysis of apoptosis, as determined by the flow cytometric evaluation to Figs. 2b, 3a, 3b, 6b and S2A. [file 13020_2020_396_MOESM2_ESM.docx]

Fig. 2B

| C-33A | | | | | |
| --- | --- | --- | --- | --- | --- |
| 10%serum | | | serum-free | | |
| DMSO | 5 μM Rh2 | 7.5 μM Rh2 | DMSO | 5 μM Rh2 | 7.5 μM Rh2 |
| 6.59±1.11 | 12.37±3.21 | 11.59±1.65 | 14.6±3.22 | 43.37±4.22 | 50.63±2.13 |

| HeLa | | | | | |
| --- | --- | --- | --- | --- | --- |
| 10%serum | | | 10%serum | | |
| DMSO | 5 μM Rh2 | 7.5 μM Rh2 | DMSO | 5 μM Rh2 | 7.5 μM Rh2 |
| 4.43±0.86 | 1.48±1.10 | 6.03±0.89 | 9.41±1.21 | 46.61±1.99 | 49.30±2.1 |

Fig. 3A

| HeLa | | | |
| --- | --- | --- | --- |
| DMSO | | AC-DEVD-CHO | |
| DMSO | 5 μM Rh2 | DMSO | 5 μM Rh2 |
| 12.73±2.11 | 40.30±2.30 | 5.08±1.32 | 39.44±2.12 |

| C-33A | | | |
| --- | --- | --- | --- |
| DMSO | | AC-DEVD-CHO | |
| DMSO | 5 μM Rh2 | DMSO | 5 μM Rh2 |
| 6.76±1.35 | 29.08±2.99 | 6.87±2.66 | 28.04±5.21 |

Fig. 3B

| HeLa | | | |
| --- | --- | --- | --- |
| 10%serum | | 10%serum | |
| DMSO | 5 μM Rh2 | DMSO | 5 μM Rh2 |
| 209578.29±6562.12 | 195522.08±5325.21 | 143898.95±8155.63 | 55945.61±7439.22 |

| C-33A | | | |
| --- | --- | --- | --- |
| 10%serum | | 10%serum | |
| DMSO | 5 μM Rh2 | DMSO | 5 μM Rh2 |
| 100435±2639.32 | 100470.95±6510.33 | 98028.57±8268.63 | 58330.32±7459.23 |

Fig. 6B

| HeLa | | | |
| --- | --- | --- | --- |
| 10%serum | | Serum-free | |
| Control siRNA | Atg7 siRNA | Control siRNA | Atg7 siRNA |
| 3.85±1.22 | 7.05±0.85 | 4.7±0.99 | 37.44±2.98 |

| C-33A | | | |
| --- | --- | --- | --- |
| 10%serum | | Serum-free | |
| Control siRNA | Atg7 siRNA | Control siRNA | Atg7 siRNA |
| 6.35±0.88 | 6.73±0.89 | 6.92±0.21 | 35.85±1.39 |

Fig. S2

| C-33A | | | |
| --- | --- | --- | --- |
| 10%serum | | Serum-free | |
| Control siRNA | Atg7 siRNA | Control siRNA | Atg7 siRNA |
| 6.35±0.88 | 6.73±0.89 | 6.92±0.21 | 35.85±1.39 |

| DMSO | | | | 5 μM Rh2 | | | |
| --- | --- | --- | --- | --- | --- | --- | --- |
| DMSO | Rapamycin | BA1 | 3-MA | DMSO | Rapamycin | BA1 | 3-MA |
| 11.68±0.65 | 11.58±0.56 | 18.40±0.35 | 65.77±1.25 | 53.84±0.86 | 38.01±1.36 | 72.37±1.32 | 74.02±1.69 |

| C-33A | | | |
| --- | --- | --- | --- |
| 10%serum | | Serum-free | |
| Control siRNA | Atg7 siRNA | Control siRNA | Atg7 siRNA |
| 6.35±0.88 | 6.73±0.89 | 6.92±0.21 | 35.85±1.39 |

| C-33A | | | |
| --- | --- | --- | --- |
| 10%serum | | Serum-free | |
| Control siRNA | Atg7 siRNA | Control siRNA | Atg7 siRNA |
| 6.35±0.88 | 6.73±0.89 | 6.92±0.21 | 35.85±1.39 |

| HeLa | | | |
| --- | --- | --- | --- |
| 10%serum | | Serum-free | |
| Control siRNA | Atg7 siRNA | Control siRNA | Atg7 siRNA |
| 3.85±1.22 | 7.05±0.85 | 4.7±0.99 | 37.44±2.98 |

| HeLa | | | |
| --- | --- | --- | --- |
| 10%serum | | Serum-free | |
| Control siRNA | Atg7 siRNA | Control siRNA | Atg7 siRNA |
| 3.85±1.22 | 7.05±0.85 | 4.7±0.99 | 37.44±2.98 |

| HeLa | | | |
| --- | --- | --- | --- |
| 10%serum | | Serum-free | |
| Control siRNA | Atg7 siRNA | Control siRNA | Atg7 siRNA |
| 3.85±1.22 | 7.05±0.85 | 4.7±0.99 | 37.44±2.98 |

| HeLa | | | |
| --- | --- | --- | --- |
| 10%serum | | Serum-free | |
| Control siRNA | Atg7 siRNA | Control siRNA | Atg7 siRNA |
| 3.85±1.22 | 7.05±0.85 | 4.7±0.99 | 37.44±2.98 |
