# Supplementary material for: Ginsenoside Rh2 represses autophagy to promote cervical cancer cell apoptosis during starvation
Source: Chin Med. 2020 Nov 12;15:118. doi: 10.1186/s13020-020-00396-w (PMC7661217; doi:10.1186/s13020-020-00396-w)
Supplement: Supplementary file 3 — Additional file 3. Supplement of materials and methods. [file 13020_2020_396_MOESM3_ESM.docx]

**Materials and Methods**

**Reagents**

Rd and Rb1 (purity>98%) were purchased from Chengdu Must Biotech (Chengdu, China). 3-Methyladenine (3-MA) was purchased from InvivoGen (San Diego, CA, USA).

**Cells and transfection**

A549 (ATCC, CCL-185), B16 (ATCC, CRL-6475) cells were cultured in DMEM supplemented with 10% fetal bovine serum (FBS) at 37°C in a 5% CO_2_ environment.

**Cell cycle measurement**

Cells were fixed with chilled 70% ethanol and stored at −20°C overnight. The next day, the cells were stained with PI for 30 min before flow cytometry. Samples were analyzed by Flow Cytometer and software FlowJo.
